# The effect of preferred background music on task-focus in sustained attention

**DOI:** 10.1007/s00426-020-01400-6

**Published:** 2020-08-03

**Authors:** Luca Kiss, Karina J. Linnell

**Affiliations:** Department of Psychology, Goldsmiths University of London, London, UK

## Abstract

Although many people listen to music while performing tasks that require sustained attention, the literature is inconclusive about its effects. The present study examined performance on a sustained-attention task and explored the effect of background music on the prevalence of different attentional states, founded on the non-linear relationship between arousal and performance. Forty students completed a variation of the *Psychomotor Vigilance Task*—that has long been used to measure sustained attention—in silence and with their self-selected or preferred music in the background. We collected subjective reports of attentional state (specifically mind-wandering, task-focus and external distraction states) as well as reaction time (RT) measures of performance. Results indicated that background music increased the proportion of task-focus states by decreasing mind-wandering states but did not affect external distraction states. Task-focus states were linked to shorter RTs than mind-wandering or external distraction states; however, background music did not reduce RT or variability of RT significantly compared to silence. These findings show for the first time that preferred background music can enhance task-focused attentional states on a low-demanding sustained-attention task and are compatible with arousal mediating the relationship between background music and task-performance.

## Introduction

Although many people listen to background music during tasks that require sustained attention, there is still no consensus about its effect on performance (for reviews, see Kämpfe, Sedlmeier, & Renkewitz, 2011; Küssner 2017). Research has focused on background music and sustained attention for decades (e.g., Davies, Lang, & Shackleton, 1973) but findings are contradictory, with some studies suggesting a positive influence of the music (e.g., Davies et al., 1973; Corhan & Gounard, 1976; Fontaine & Schwalm, 1979; Turner, Fernandez, & Nelson, 1996; Ünal, Waard de, Epstude, & Steg, 2013) but others suggesting the opposite (e.g., Brodsky & Slor, 2013; Febriandirza, Wu, Ming, Hu, & Zhang, 2017; North & Hargreaves, 1999; Shih, Huang, & Chiang, 2012).

**Fig. 1:**
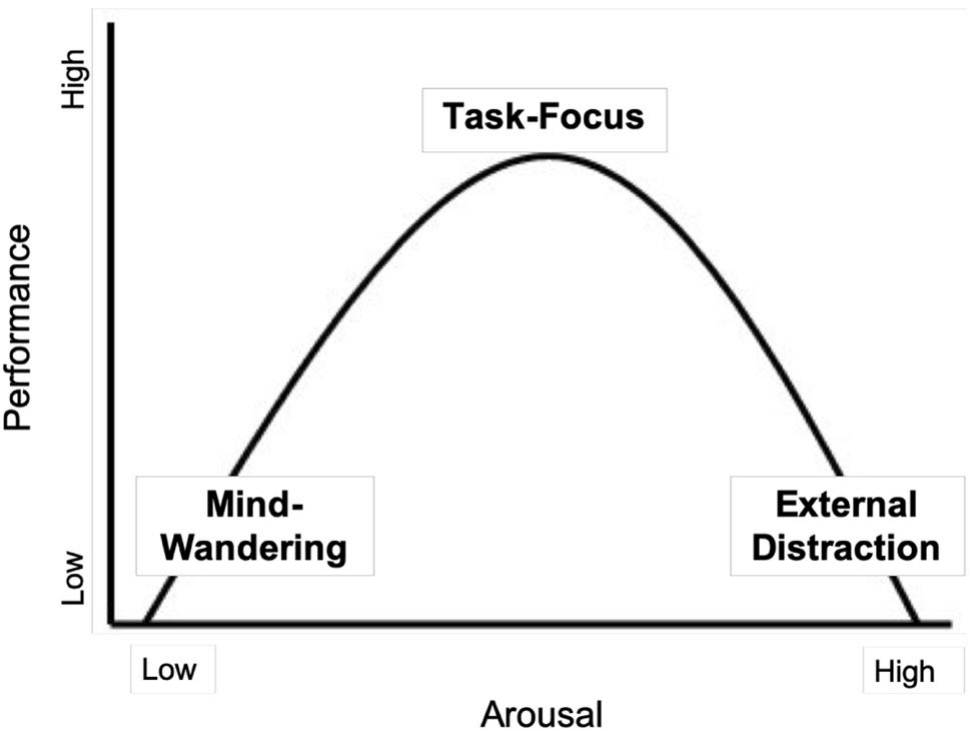
Inverted-U relationship between arousal, attentional states (mind-wandering, task-focus, external distraction) and performance, as hypothesised by Unsworth and Robison (2016) and in similar form Yerkes and Dodson (1908)

Even though sustained attention is crucial for successful performance (Robertson & O’Connell, 2010), sustaining focus on task-relevant information over an extended time period is demanding, leading to time-on-task effects and attentional lapses (Robertson, Manly, Andrade, Baddeley, & Yiend, 1997; Unsworth & Robison, 2016). Attentional lapses have been found to be underpinned by the locus coeruleus–norepinephrine system (LC–NE; Cohen, Aston-Jones, & Gilzenrat, 2004), a neuromodulatory nucleus in the brain stem that projects norepinephrine to the neocortex and mediates effects of arousal (Berridge & Waterhouse, 2003). When baseline LC activity, or arousal, is either too low or too high, people perform poorer (Aston-Jones & Cohen, 2005) and experience attentional lapses that have been characterised, respectively, as episodes of mind-wandering (hypo-arousal) or external distraction (hyper-arousal) (i.e., off-task attentional states; Unsworth & Robison, 2016). Only an intermediate baseline LC activity is linked to optimal performance and a task-focused attentional state (i.e., on-task state; see Fig. 1; Aston-Jones & Cohen, 2005; Unsworth & Robison, 2016).

Understanding the effect of background music on these different arousal-driven on-task and off-task attentional states (see Fig. 1 above) is important for theoretical as well as practical reasons: it is important for improving our understanding both (i) of the real-life situations in which background music listening might be beneficial for attention and performance and also (ii) of core attentional function, and the complex relationship between arousal and attention. To our knowledge, our study is the first that has explored the relationship between background music and the prevalence of different attentional states—both on-task and off-task—on a sustained-attention task. In addition, the fact that it focused on self-selected background music distinguishes it further and gives it ecological validity and contemporary relevance.

As previous research has suggested, arousal may mediate the relationship between background music and performance (Beh & Hirst, 1999; Cassidy & MacDonald, 2007; North & Hargreaves, 1999; Ünal et al., 2013; Wang, Jimison, Richard, & Chuan, 2015). Specifically, in line with the inverted-U curve linking arousal to performance, previous research has found that background music increases arousal (Burkhard, Elmer, Kara, Brauchli, & Jäncke, 2018; Ünal et al., 2013) and that, when people are presented with an easy low-arousing task, the music can increase arousal resulting in better performance (Fontaine & Schwalm, 1979; Ünal et al., 2013). Contrarily, when people are presented with a difficult more arousing task, the music results in poorer performance, compatible with music increasing arousal to too high a level (Beh & Hirst, 1999; North & Hargreaves, 1999; Wang et al., 2015). In the current study, we used an easy low-arousing sustained-attention task—a version of the standard sustained-attention task called the Psychomotor Vigilance Task previously used by Unsworth and Robison (2016)—to imitate the boringness of real-life monotonous tasks and to maximise the potential for music to produce, via arousal, a beneficial effect.

Using self-selected or preferred music in this study, we could manipulate the ‘arousingness’ of the environment in an ecologically valid manner and control for personal preferences (Cassidy & MacDonald, 2009; Darrow, Johnson, Agnew, Fuller, & Uchisaka, 2006; Ünal, Steg, & Epstude, 2012, Ünal et al.,  2013) as well as individual differences in baseline arousal. Because baseline arousal level varies across people and depends on factors, such as personality (e.g., Cassidy & MacDonald, 2007; Furnham & Allass, 1999; Salame & Baddeley, 1982), the ‘arousingness’ and the qualities of the music required to achieve optimal arousal and performance also varies across people. Nevertheless, we conducted exploratory analyses of the impact on sustained attention of qualities of the preferred music stimuli, namely tempo, lyrics and genre, as these have all been shown to impact attention and performance (see, e.g., Amezcua, Guevara, & Ramos-Loyo, 2005; Angel, Polzella, & Elvers, 2010; Chew, 2010; Corhan & Gounard, 1976; Darrow et al., 2006; Davies et al., 1973; Drai-Zerbib & Baccino, 2017; Shih et al., 2012).

In this study, we aimed to see how preferred background music impacts the distribution of attentional states, as well as the effects of time-on-task on sustained attention. By adopting subjective reports of attentional states, the present study aimed to provide a richer and more informative measure of attention than is available from performance-based measures (Unsworth & Robison, 2016). Participants were asked to report their conscious experience at random periods throughout the task. Eliciting these subjective reports allowed us to distinguish poorer performance that resulted from the two types of attentional lapses, mind-wandering (hypo-arousal) and external distraction (hyper-arousal), and to explore the differential effect of background music on these lapses. Furthermore, measuring the percentage of task-focus reports gave an indication of how close to optimal performance was, in a way that focusing only on performance-based measures could not.

In addition to collecting subjective reports of attentional state, the present study examined the impact of background music on time-on-task effects. Background music might exert a positive influence on time-on-task effects via arousal: given that arousal falls off with time-on-task (e.g., Whyte, Polansky, Fleming, Coslett, & Cavallucci, 1995), it might need to be boosted by the music over time to reach an optimal level. Alternatively, it could also be the case that any beneficial effects of the music over time would be counteracted by the increasing tendency of participants to habituate to the music (Burkhard et al., 2018).

In our first hypothesis, we predicted an interaction effect between attentional-state category (i.e., mind-wandering, task-focus, external distraction) and background music. Specifically, we expected that preferred background music would increase the proportion of task-focus states compared to states of mind-wandering and external distraction. This interaction effect was measured on proportions of subjectively reported attentional states as a function of music-present/absent. We also looked at how the proportion of task-focus reports changes during performance as a measure of time-on-task.

In the second hypothesis, we expected on-task states (task-focus) to be linked to faster reaction times (RTs) than off-task states (mind-wandering, external distraction) to verify that subjective reports are a valid representation of attentional state and express themselves in behaviour (Unsworth & Robison, 2016).

Our third hypothesis was based on the first two hypotheses and predicted that if background music is linked to more task-focus states and task-focus is linked to faster and less variable RTs, then we would expect an effect of background music on RTs. Therefore, we hypothesised that mean RTs would be shorter, and increases in RTs with time-on-task would be smaller, with background music than in silence.

## Methods

### Design

The design was a within-subject design; all participants completed the task in silence and with background music. The independent variables were music-present/absent, time-on-task (block 1, 2, 3, 4, and 5) and attentional-state category (mind-wandering, task-focus, external distraction). The order of the music conditions was counterbalanced, meaning that every second, participant completed the task in the same order. This resulted in 20 participants completing the task in music-present followed by music-absent order and 20 participants in music-absent followed by music-present order. One dependent variable was the frequency with which each of the three attentional-state categories was reported, henceforth termed thought-probe response proportions. The other two dependent variables were RT (in milliseconds; ms), and standard deviation of RT, while performing a sustained-attention task. Prior to the main experiment, a pilot study was conducted.

### Participants

#### Main study

Participants were students living in and around London, who took part in the study on a voluntary basis in exchange for £5. Only students who normally listen to background music when performing attention-demanding tasks were included in the experiment. This inclusion criterion was supported by findings that people perform better in their preferred listening condition (Crawford & Strapp, 1994; Nantais & Schellenberg, 1999). There were 40 participants in total. The sample size was based on a power calculation showing that a minimum sample of 34 people would be sufficient for a medium (0.25) effect size with a 0.80 power level and 0.05 alpha level.

Participants included 23 females and 17 males between the ages of 19 and 32 (*M* = 24, *SD* = 3.33). Analysis of gender did not show any significant effects on thought-probe response proportions or on overall RT measures of performance, nor a significant interaction with music-present/absent on these measures on the sustained-attention task. Thus, gender is not included in further analyses of these measures. This null result agrees with past research suggesting that there is no effect of gender on sustained attention (e.g., Chan, 2009).

27 of the participants had received previous formal musical training (voice or instrument) in a music school or private setting (*M* = 6 years; Min. = 2 years; Max. = 21 years). Musical training was measured by asking participants whether they had had any previous musical training and if so, for how many years. Analysis of musical training did not show any significant effects on thought-probe response proportions or on overall RT measures of performance, nor a significant interaction with music-present/absent on these measures. Therefore, musical training is not analysed further here. Indeed, although there are previous studies that have shown an effect of musical training on sustained attention, they have not shown an interaction between training and the effect of background music (e.g., Darrow et al., 2006; Rodrigues, Loureiro, & Caramelli, 2013).

#### Pilot study

A pilot study was conducted to refine the thought-probe stimuli (see “Materials”). A different set of ten participants completed the pilot study, seven females and three males, who were all students at London-based universities.

### Materials

#### Preferred background music

Participants were asked to send a 30-min long playlist containing their preferred background music tracks to the experimenter prior to participation in the study. There were no restrictions on the music, but participants were asked to send a playlist they would normally listen to when performing an attention-demanding task. The individual tracks could be of any length (mean track duration was 215.57 s) but together the playlist had to cover the full 30 min of the music-present session of the experiment. A few playlists were shorter than 30 min, because some participants decided to listen to their chosen tracks in repeat, as they would normally do in real-life settings. Musicological data for each track were collected from the database of the digital music streaming service called Spotify, using the Spotify Web API endpoints (Spotify 2018). This included data on tempo (overall tempo of a track in beats/minute; *M* = 112.56 BPM, *SD* = 32.37 BPM, range for individual tracks = 52.16–215.04 BPM), lyrics (i.e., a measure between 0.00 and 1.00 indicating the likelihood of a track containing any vocals at any given point during the track; tracks with a lower lyrics level were less likely to contain any vocal content; *M* = 0.59, *SD* = 0.57, range for individual tracks = 0.01–1.00), and genre. Genre categorisation for each track was based on Spotify’s main genre categories (Spotify 2018); because there was no specific genre categorisation available for individual tracks, genres describing the artist and the album in which the track appeared were used. Specifically, the most frequently occurring main genre was chosen for each track. Tracks that only had subgenre labels and not a clear main genre assigned to them were placed in the ‘other’ category.

Although some previous studies have shown that music preferences are influenced by gender, suggesting that males prefer “heavier” (Colley, 2008), more “vigorous” (Soares-Quadros Júnior, Lorenzo, Herrera, & Araújo Santos, 2019), and “intense-rebellious” music (Dobrota, Reić Ercegovac, & Habe, 2019) compared to females, in this study, there was no effect of gender on preferences for either tempo, lyrics, or genre.

#### Sustained attention task

**Fig. 2:**
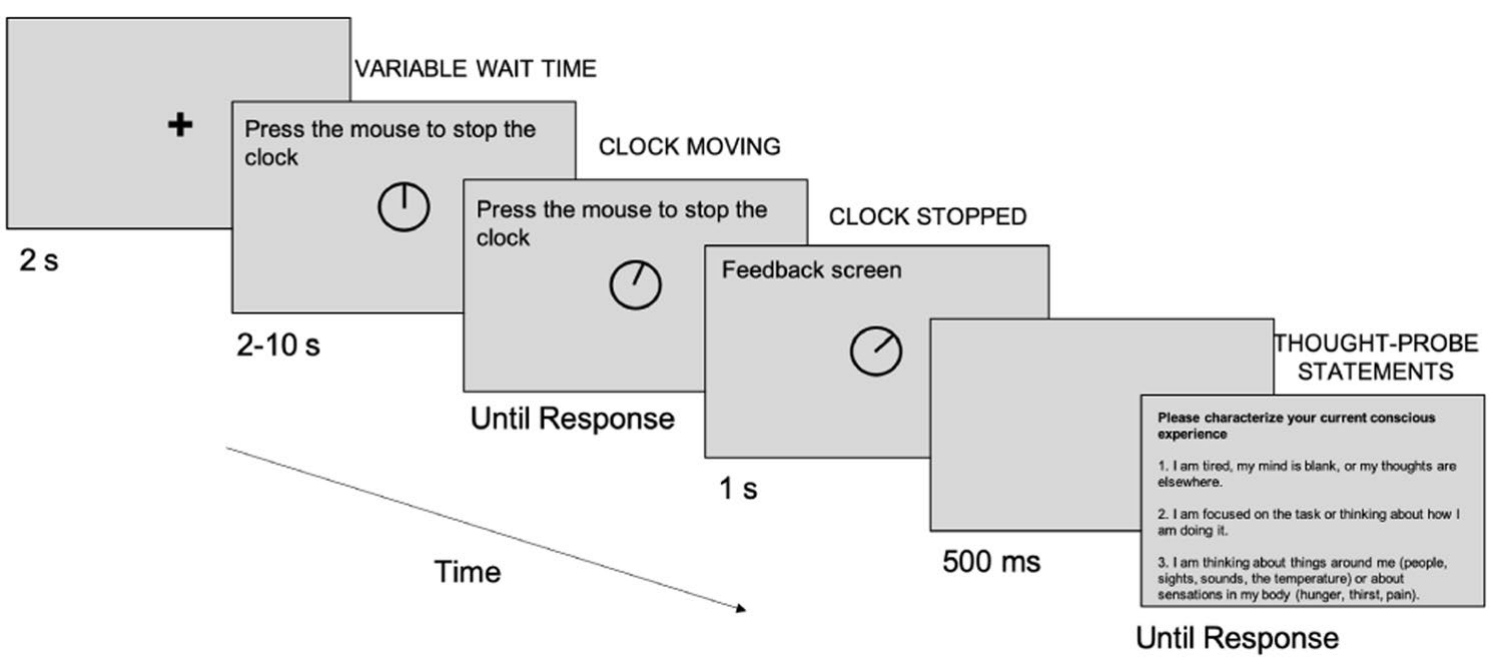
Schematic representation of a single experimental trial with a thought-probe after the trial

The sustained-attention task was a variation of the Psychomotor Vigilance Task developed by Dinges and Powell (1985), which has long been used to measure sustained attention (Unsworth & Robison, 2016). As shown in Fig. 2, participants were first presented with a fixation cross in the middle of the screen on a grey background for 2 s (s). Then, they saw a clock without any numbers, specifically a black circle containing a clock-hand at the 12-o’clock position and, after a variable wait time (equally distributed from 2 to 10 s in 500 ms increments), the clock-hand started moving clockwise in a smooth analogue fashion. The analogue clock was developed instead of the digital one used in previous experiments to make the task both applicable with populations with different/no number systems and also lower in cognitive demand. The task of the participants was to press the left mouse button as quickly as possible once the hand of the clock started moving. After the participant had pressed the mouse button, the clock remained on the screen for 1 s to provide feedback. Then, a 500-ms blank screen was presented, followed by either the next trial or a thought-probe. Participants completed 5 blocks, with 34 trials in each block, for both the music and no-music conditions. One block lasted approximately 6 min. Before starting the experiment, participants completed five practice trials to become familiar with the task.

During each block, participants were periodically presented with thought-probes. They were primed to respond to these probes by providing subjective reports to classify their immediately preceding thoughts. In all, they were presented with six thought-probes per block, after six randomly selected trials.

Thought-probes were refined in the pilot study based on participants’ feedback. Data obtained in the pilot study phase were not analysed or reported. Thought-probe statements, as specified below, were based on those used by Unsworth and Robison (2016). The original statements that were used by Unsworth and Robison (2016) are presented below:

“Please characterise your current conscious experience.

1. I am totally focused on the current task.
2. I am thinking about my performance on the task or how long it is taking.
3. I am distracted by sights/sounds/temperature or by physical sensations (hungry/thirsty).
4. I am daydreaming/my mind is wandering about things unrelated to the task.
5. I am not very alert/my mind is blank or I’m drowsy.”

Some alterations were made compared to previous research based on feedback from the participants in the pilot study, to avoid ambiguous expressions that could refer to different concepts depending on one’s cultural background and understanding. More specifically, compared to the original five statements, only three statements were included in this study, namely those referring to mind-wandering, on-task thoughts/task-focus and external distraction, respectively (see the statements below). The term mind-wandering was left out of the phrasing of statement 1, and statements 2 and 3—referring to task-focus and external distraction—were simplified.

“Please characterise your current conscious experience.

1. I am tired, my mind is blank, or my thoughts are elsewhere.
2. I am focused on the task or how I am doing it.
3. I am thinking about the things around me (people, sights, sounds, the temperature) or about sensations in my body (hunger, thirst, pain).”

### Apparatus and procedure

The present study was approved by the Psychology Department Ethics Committee at Goldsmiths, University of London on the 6th of June 2018. Participants were tested individually in the lab, in a dark and quiet room, and stimuli were presented on a 24-in. monitor with a 1920 × 1080 screen resolution.

Participants were asked not to do any heavy exercise or drink any caffeinated drinks 2–3 h prior to participation and to have a good night’s sleep involving at least 7–8 h sleep the night before. Prior to the day of the study, participants sent a 30-min long playlist with their preferred background tracks to the researcher. Background music was played continuously throughout the task in the music condition from a mobile device in “do not disturb” mode to avoid any distractions. Participants could either use their own headphones or a pair provided by the researcher to listen to the music. For the music-absent condition, the headphones were removed to increase ecological validity (given that participants would not normally wear headphones when completing a task in silence).

Upon first arrival in the lab at the start of the experiment, participants first read the information sheet and signed the consent form. Then, they received the task instructions and were presented with five practice trials before the first block in both the music-present and music-absent conditions. They had the opportunity to ask any questions throughout the practice trials.

The instructions they received for the practice trials also applied to the main task. First participants were instructed to look at the fixation cross in the centre of the screen before each trial. Then, it was explained to them that their tasks were (i) to stop the clock-hand in the clock-face in the middle of the screen as soon as the clock-hand started moving, by pressing the left mouse button; (ii) when presented with thought-probe statements after some of the trials, to choose the statement that best described their current conscious experience by pressing buttons 1, 2, or 3 on the keyboard.

The study took approximately 1 h and 30 min, during which time participants performed the sustained-attention task twice: once in silence (30 min) and once with their preferred music playing in the background (30 min). There were five blocks of trials in each condition. Each block was started individually by the experimenter, ensuring that there was a break for a few minutes between the blocks so that participants could rest and drink some water if they wished to. To control for carry-over effects of the music conditions, music conditions were counterbalanced and there was a 10-min break between the conditions in both orders. During the break, participants used a tablet to play a word spelling game called ‘Hi Words’ that was unrelated to the study. The game kept them engaged and helped them recover from the fatigue caused by the first condition (e.g., Jahncke, Hygge, Halin, Green, & Dimberg, 2011).

Once participants had finished the task, the researcher recorded their age, gender and whether they had had any previous formal music training and, if yes, for how many years. Then, the participants received the debriefing sheet and £5 for their participation.

## Results

Analyses were conducted on the effect of preferred background music both on the subjective measures of attention and behavioural measures of performance. The independent variables were music-present/absent, time-on-task (blocks 1, 2, 3, 4, and 5) and attentional-state category (mind-wandering, task-focus, external distraction). For the dependent variables, following Unsworth and Robison (2016), we examined: thought-probe response proportions of attentional-state category, mean RT (in ms) and standard deviation of RT (in ms). For the two RT analyses, some data points were removed, including any number below 100 ms (18 data points) based on Basner and Dinges’ (2011) suggestion that RTs below 100 ms count as false starts (errors of commission); moreover, any number above 5000 ms that exceeded the time required for the clock-hand to complete a full revolution was also removed (two data points).

Order of the music conditions (music-absent followed by music-present, or music-present followed by music-absent) did not show any significant effects on thought-probe response proportions, RT, or standard deviation of RT (*p* ≥ 0.23), and there was no interaction between order and music-present/absent on these variables (*p* ≥ 0.10); therefore, analyses were collapsed across order.

In all, four different analyses were conducted on thought-probe response proportions, RT, and standard deviation of RT. In the first analysis, a three-way repeated-measures ANOVA was conducted to examine the effect of attentional-state category, music-present/absent and time-on-task on thought-probe response proportions. In the second analysis, RT was analysed; however, based on Unsworth and Robison (2016), we anticipated having insufficient data to perform the corresponding three-way ANOVA. In other words, we anticipated having too many empty cells for RT response for trials associated with thought-probe statements as a consequence of participants not all reporting all attentional-state categories. Therefore, a separate linear-mixed-model analysis was used, which accounted for unequal sample sizes, to explore the effect of attentional-state category on RT as a check that the subjective reports are meaningful and express themselves in behaviour. The third analysis also focused on RT: it was a two-way repeated-measures ANOVA and was performed to analyse the effect of music presence/absence and time-on-task on RT. Finally, the fourth analysis focused on the standard deviation of RT: like the third, it was a two-way repeated-measures ANOVA conducted on the effect of music-present/absent and time-on-task, but this time on the standard deviation of RT.

At the end of the Results section, we report additional analyses on tempo, lyrics, and genre. These additional analyses should be treated as exploratory given that participants were free to select tracks that varied in tempo, lyrics, and genre. As a result, determining the values of these musicological parameters for each participant involved factoring in the proportion of time that the participant spent listening to tracks of varying tempo, lyrics, and genre.

### The effect of music-present/absent, attentional-sate category, and time-on-task on thought-probe response proportions

To analyse the effect of background music on thought-probe response proportions, a 2 × 3 × 5 repeated-measures factorial ANOVA was conducted on music-present/absent, attentional-state category and time-on-task. Greenhouse–Geisser correction was used, because the sphericity assumption was violated, *p* < 0.02. There was a significant main effect of attentional-state category, *F*(1.66, 64.98) = 35.06, *MSE* = 21.64, *p* < 0.001, partial *η*^2^ = 0.47, and a significant quadratic trend, *F*(1, 39) = 50.97, *MSE* = 35.98, *p* < 0.001, partial* η*^2^ = 0.57. This shows that participants reported significantly more task-focus states (*M* = 0.58, *SD* = 0.33) than mind-wandering (*M* = 0.22, *SD* = 0.28) or external distraction states (*M* = 0.20, *SD* = 0.24).

**Fig. 3:**
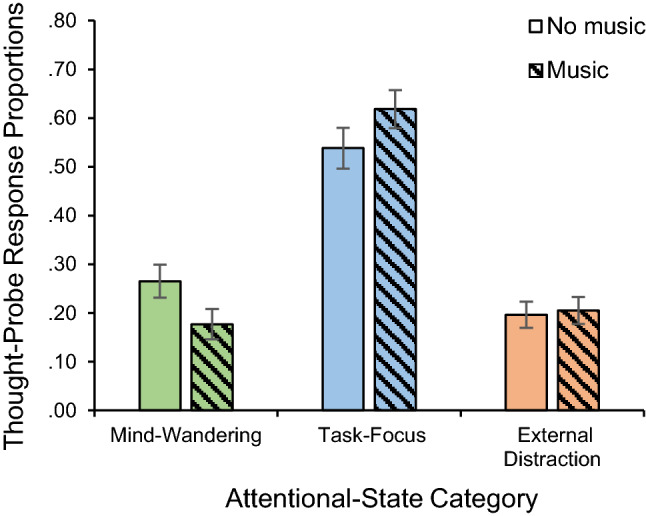
Thought-probe response proportions as a function of attentional-state category and music-present/absent (music/no music). Error bars represent ± 1 standard error of the mean (S.E.M.)

Importantly, there was also a significant interaction between music-present/absent and attentional-state category, *F*(1.45, 56.37) = 4.03, *MSE* = 0.98, *p* = 0.04, partial* η*^2^ = 0.09, and a significant linear trend, *F*(1, 39) = 6.44, *MSE* = 0.46, *p* = 0.02, partial* η*^2^ = 0.14. The simple effect analysis (paired-sample *t* tests) showed a significant difference between music-present and music-absent conditions in the proportion of both mind-wandering, *t*(199) = 4.24, *p* < 0.001, *d* = 0.30, and task-focus reports, *t*(199) = − 2.82, *p* = 0.005, *d* = 0.20, and a non-significant difference in external distraction reports, (*p* = 0.72, *d* = 0.03). Specifically, there were significantly more mind-wandering reports in the music-absent condition (*M* = 0.27, *SD* = 0.28) than in the music-present condition (*M* = 0.18, *SD* = 0.28) and significantly more task-focus reports in the music-present condition (*M* = 0.62, *SD* = 0.34) than in the music-absent condition (*M* = 0.54, *SD* = 0.33), as seen in Fig. 3.

**Fig. 4:**
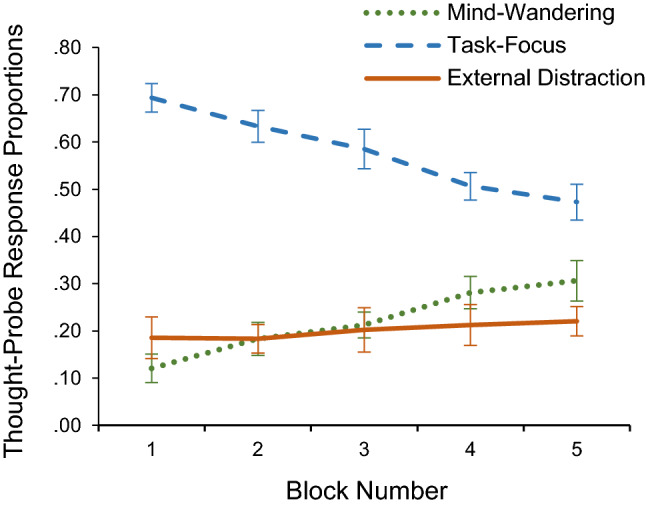
Thought-probe response proportions as a function of time-on-task (block number). Error bars represent ± 1 S.E.M

Furthermore, the ANOVA also revealed a significant interaction between attentional-state category and time-on-task, *F*(5.00, 195.13) = 8.23, *MSE* = 0.90, *p* < 0.001, partial* η*^2^ = 0.17, and a significant linear, *F*(1, 39) = 5.81, *MSE* = 0.55, *p* = 0.02, partial* η*^2^ = 0.13, and quadratic trend, *F*(1, 39) = 25.05, *MSE* = 3.87, *p* < 0.001, partial* η*^2^ = 0.39 (see Fig. 4).

The simple effect analysis (using paired-sample t tests with Bonferroni correction) on mind-wandering reports and time-on-task showed a significant difference in the proportion of mind-wandering reports between blocks 1–3 (*p* = 0.003, *d* = 0.49), 1–4 (*p* < 0.001, *d* = 0.72), 1–5 (*p* < 0.001, *d* = 0.62), 2–4 (*p* = 0.001, *d* = 0.57), and 2–5 (*p* = 0.006, *d* = 0.46). A simple regression analysis demonstrated that, as block number increased, the proportion of mind-wandering reports also increased, *F*(1, 3) = 192.00, *p* = 0.001, *R*^2^ = 0.99.

Moreover, the simple effect analysis (using paired-sample t tests with Bonferroni correction) on task-focus reports and time-on-task also showed a significant difference in the proportion of task-focus reports between blocks 1–3 (*p* = 0.007, *d* = 0.45), 1–4 (*p* < 0.001, *d* = 0.71), 1–5 (*p* < 0.001, *d* = 0.70), 2–4 (*p* < 0.001, *d* = 0.60), 2–5 (*p* < 0.001, *d* = 0.64), 3–4 (*p* = 0.004, *d* = 0.48) and 3–5 (*p* = 0.005, *d* = 0.47). A simple regression analysis demonstrated that, as block number increased, the proportion of task-focus reports decreased, *F*(1, 3) = 294.00, *p* < 0.001, *R*^2^ = 0.99.

The simple effect analysis (paired-sample *t* tests) on external distraction reports and time-on-task did not show any significant difference in the proportion of external distraction reports between blocks (*p* ≥ 0.23, *d* ≤ 0.19).

Finally, the ANOVA revealed that there was not a significant 3-way interaction between music-present/absent, attentional-state category and time-on-task *(p* = 0.13).

### The effect of attentional-sate category on reaction time

Because not all participants reported all of the attentional states, there were too many missing cases to perform a corresponding (three-way ANOVA) analysis on RT. Therefore, a separate linear-mixed-model analysis was performed on attentional-state category and RT, followed by a two-way ANOVA on the effect on RT of time-on-task and music-present/absent. Finally, a similar two-way ANOVA was performed on the effect of time-on-task and music-present/absent on the standard deviation of RT.

**Fig. 5:**
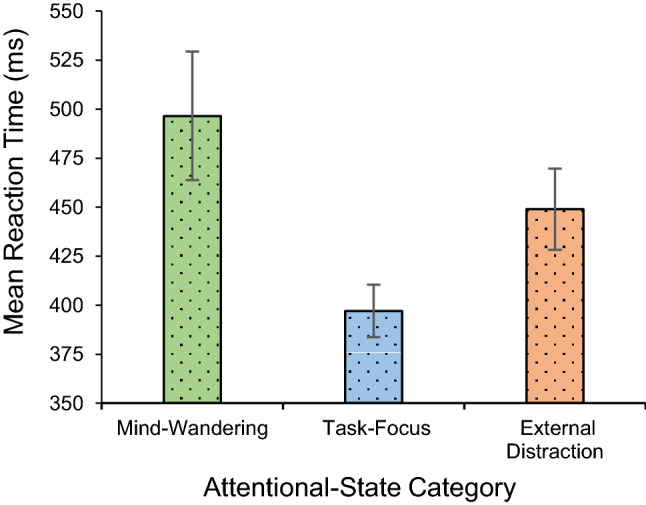
Mean RT as a function of attentional-state category. Error bars represent ± 1 S.E.M

First, the relationship between attentional-state category and RT was examined to explore the correlation between subjective and behavioural measures. We expected that on-task (task-focus) states would be linked to faster reaction time than off-task states (mind-wandering and external distraction). Because four participants did not report mind-wandering and sample sizes were unequal (40 participants in the task-focus group, 40 in the external-distraction group, and 36 participants in the mind-wandering group), we performed linear-mixed-model analysis following Unsworth and Robison (2016). In the model, attentional-state category was entered as a fixed factor and subjects were entered as random factors. The linear-mixed-model analysis suggested that on-task reports were associated with significantly shorter RTs than mind-wandering reports, *t*(35.48) = 3.89, *p* < 0.001 (*b* = 29.61, *SE* = 13.41), and also with significantly shorter RTs than external distraction reports, *t*(39) =  − 4.39, *p* < 0.001 (*b* = 21.71, *SE* = 20.68) (see Fig. 5).

### The Effect of music-present/absent and time-on-task on reaction time

**Fig. 6:**
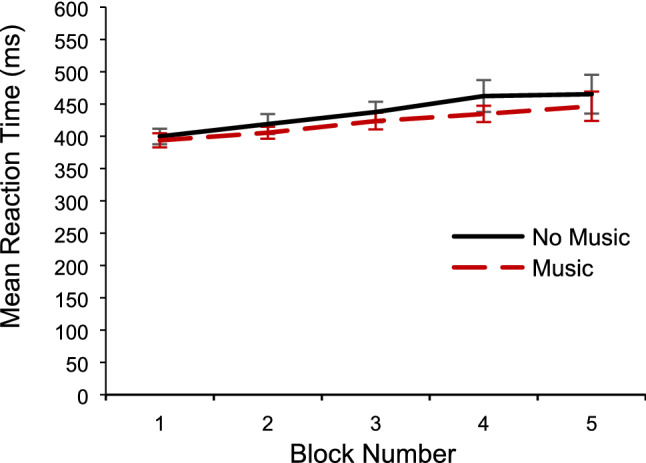
Mean RT as a function of time-on-task (block number) and music-present/absent (music/no music). Error bars represent ± 1 S.E.M

Next, a 5 × 2 repeated-measures ANOVA was conducted on the effect of music-present/absent and time-on-task on RT. Greenhouse–Geisser correction was used for the effect of time-on-task, because the sphericity assumption was violated, *p* < 0.001. There was a significant main effect of time-on-task, *F*(1.44, 56.06) = 7.64, *MSE* = 17,793.34, *p* = 0.003, partial* η*^2^ = *0.1*6, and a significant linear trend of time-on-task, *F*(1, 39) = 9.90, *MSE* = 19,464.16, *p* = 0.003, partial* η*^2^ = *0.2*0. This means that RT became significantly slower as block number increased (see Fig. 6).

The ANOVA did not show a significant main effect of music-present/absent (*p* = 0.12), nor a significant interaction between music-present/absent and time-on-task (*p* = 0.46).

### The Effect of music-present/absent and time-on-task on standard deviation of RT

**Fig. 7:**
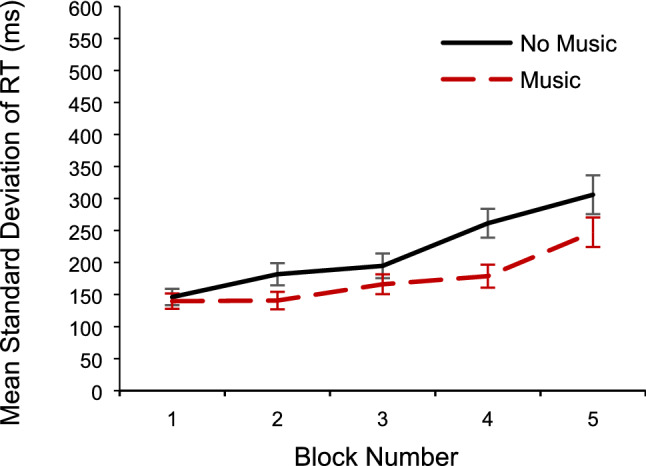
Standard deviation of RT as a function of time-on-task (block number) and music-present/absent (music/no music). Error bars represent ± 1 S.E.M

In addition to analysing RTs, the standard deviations of RTs were also analysed in a separate 5 × 2 repeated-measures ANOVA on music-present/absent and time-on-task. Greenhouse–Geisser correction was used for the effect of time-on-task, because the sphericity assumption was violated *p* < 0.001. There was a significant main effect of time-on-task, *F*(2.01, 78.45) = 4.47, *MSE* = 19,177.63, *p* = 0.01, partial* η*^2^ = *0.1*0, and a significant linear trend of time-on-task, *F*(1, 39) = 7.20, *MSE* = 164,885.83, *p* = 0.01, partial *η*^2^ = *0.1*6. This suggests that not only did participants get slower across blocks, but they also became more variable in their responding as block number increased (see Fig. 7).

The ANOVA did again not show a significant main effect of music-present/absent (*p* = 0.07), nor a significant interaction between music-present/absent and time-on-task (*p* = 0.17).

### The Effect of music tempo, lyrics, and genre on thought-probe response proportions, RT, and standard deviation of RT

**Fig. 8:**
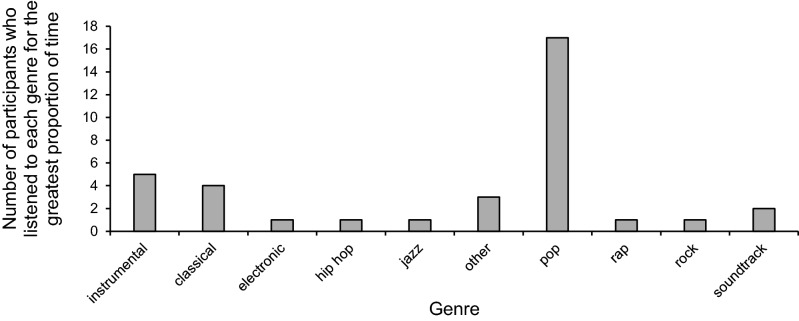
Each participant was assigned to the genre that made up the greatest proportion of time in their playlist. The graph represents the number of participants for each genre who listened to that genre for the greatest proportion of time. The ‘other’ category included tracks that could not be otherwise categorised, such as Japanese anime music, music for meditation, and some indie and folk tracks that did not clearly fit into a different main category. The ‘instrumental’ category included tracks that were primarily produced using musical instruments, such as remixes of original tracks, piano ‘lounge’ music, and ‘ambient’ music

The effects of tempo, lyrics, and genre were analysed on thought-probe response proportions, on mean RT, and on standard deviation of RT. Tempo was determined for each participant using the average value across tracks, weighted by the duration of each track (*M* = 115.90 BPM, *SD* = 14.70 BPM, range = 91.32–160.02 BPM). Similarly, lyrics were determined for each participant using the average value across tracks, weighted by the duration of each track (*M* = 0.64, *SD* = 0.36, range 0.07–1.00). For genre, each participant was assigned to the genre that made up the greatest proportion of time in their playlist (Fig. 8).

The sample size for lyrics was 36 because the chosen playlists from four participants were not available on Spotify and, therefore, could not be analysed. The sample size for analyses of tempo was 35, because there was one outlier (with an average tempo of 160.02 BPM) which was excluded from the analyses. The sample size for the analyses of genre was 22, because we compared only the two most popular genres, operationalised as those that were listened to for the greatest proportion of time by the most participants (pop and instrumental).

#### Tempo

Five separate simple regression analyses were conducted to see whether tempo can predict (i) proportions of mind-wandering reports, (ii) proportions of task-focus reports, (iii) proportions of external distraction reports, (iv) mean RT scores, and (v) standard deviation of RT. The regression models did not show any association between tempo and mind-wandering states (*p* = 0.66), task-focus states (*p* = 0.32), or external distraction states (*p* = 0.36). Moreover, the regression models also did not show any association between tempo and mean RT (*p* = 0.21), or tempo and standard deviation of RT (*p* = 0.30).

#### Lyrics

Five separate simple regression analyses were conducted to see whether lyrics can predict (i) proportions of mind-wandering reports, (ii) proportions of task-focus reports, (iii) proportions of external distraction reports, (iv) mean RT scores, and (v) standard deviation of RT. The regression models did not show any association between lyrics and mind-wandering states (*p* = 0.15), task-focus states (*p* = 0.38), or external distraction states (*p* = 0.69). Moreover, the regression models also did not show any association between lyrics and mean RT (*p* = 0.98), or lyrics and standard deviation of RT (*p* = 0.08).

#### Genre

Five separate Mann–Whitney *U* non-parametric tests were conducted to see whether genre (pop, instrumental) affects (i) proportions of mind-wandering reports, (ii) proportions of task-focus reports, (iii) proportions of external distraction reports, (iv) mean RT scores, and (v) standard deviation of RT. These non-parametric tests were chosen instead of independent *t* tests because of the unequal sample sizes: there were 17 participants in the pop, and 5 in the instrumental genre category. The Mann–Whitney *U* tests did not show any effect of genre on mind-wandering reports (*p* = 0.16), task-focus reports (*p* = 0.06), or external distraction reports (*p* = 0.25). Moreover, there was no effect of genre on RT (*p* = 0.88) or standard deviation of RT (*p* = 0.14).

## Discussion

The goal of the present study was to explore how background music affects task-performance and attentional state on a simple sustained-attention task. This study was the first that has used subjective reports of attentional state (representative of the different regions of the inverted-U curve linking arousal to performance; Unsworth & Robison, 2016) to explore the effect of background music. The results provided evidence for our first and second hypotheses in which we predicted that background music would increase the proportions of task-focus states compared to silence, and on-task states (i.e., task-focus) would be linked to shorter RTs than off-task states (i.e., mind-wandering, external distraction). However, the third hypothesis was not supported by the results and background music did not affect RT.

As predicted in the first hypothesis, background music was shown to increase task-focus states compared to silence. Specifically, it was found that, while mind-wandering reports decreased, task-focus reports increased when music was playing in the background. Interestingly, all the increase in task-focus could be accounted for by the decrease in mind-wandering, and music did not affect external distraction. In addition, task-focus reports significantly decreased, and mind-wandering reports significantly increased, with time-on-task, suggesting that people were less able to sustain their attention on the task over time and their arousal dropped. These results are likely to be a consequence of the boringness of the sustained-attention task used in the current study, with the result that it made participants hypo-aroused, placing them at the lower end of the arousal axis on the inverted-U curve. As a consequence, the arousal-increase caused by the music led to an intermediate level of arousal, shifting the balance between task-focus and mind-wandering states, and not impacting external distraction states.

This is in line with explanations that the less complex the task, the more positive the influence of music can be expected to be (Anderson 1994; Gellatly & Meyer, 1992; North & Hargreaves, 1999). For example, the present finding is supported by previous research showing that performance is better with background music compared to silence on simple tasks (Ünal et al., 2013). Specifically, Ünal et al. (2013) used a monotonous lane-keeping task and showed that preferred background music increased heart rate and some aspects of driving performance (response latencies to changes in speed on the road, lateral control) compared to silence. Similarly, Fontaine and Schwalm (1979) found that, compared to unfamiliar background music and silence, familiar music increased participants’ heart rate and correct detections on a simple vigilance task.

Familiar background music has also been linked to less frequent mind-wandering episodes and faster RTs than unfamiliar music by Feng and Bidelman (2015). They explored how familiar classical and unfamiliar classical music in the background affects mind-wandering on a lexical congruity task and found that familiar music was associated with faster response times and less frequent mind-wandering episodes than unfamiliar music. Feng and Bidelman (2015) suggested that the more positive effect of familiar music could have been a result of its familiarity increasing emotional arousal and pleasure, and in turn decreasing stress. However, they did not compare the effect of music to silence, nor did they focus on a sustained-attention task (Feng & Bidelman, 2015). Because preferred music is almost always familiar to the listener, these previous results showing a positive effect of familiar background music support the potential of preferred background music to enhance task-focused attention, as found in this study.

These past findings and the present results are compatible with the idea that the effect of background music is mediated by arousal. This idea has been raised by North and Hargreaves (1999), who discussed two possible frameworks—the arousal framework and the cognitive-load framework—that could potentially explain the effect of background music on performance. In terms of the arousal framework already outlined above, when presented with a very simple task, background music should increase arousal to an optimal/intermediate level and, thus, increase performance. Contrarily, in terms of the cognitive-load framework, if the music takes up cognitive space, then it should impair performance even on very simple tasks because it decreases the cognitive space available for the task. The current study provided evidence for the arousal framework because it showed that background music improved performance, specifically by increasing the ratio of task-focus states compared to mind-wandering states which is compatible with an increase in arousal to levels that are optimal for performance.

The results presented here also provided evidence for our second hypothesis, which predicted that states of task-focus will be linked to shorter RTs than off-task states of mind-wandering and external distraction. Therefore, our study was successful in replicating the result by Unsworth and Robison (2016) showing that on-task states are associated with shorter RTs than off-task states. This result shows that there is a link between subjective and behavioural measures of attentional state and that subjective reports provide valid, meaningful measures of attentional state that express themselves in behaviour.

Our third hypothesis was the only one not supported by the results of the main analyses: background music did not exert effects on either RT or variability of RT. In other words, even though music enhanced task-focus and task-focus was linked to shorter RT, music did not produce an overall significant reduction in RT compared to silence. The predicted effects of music on RT time-on-task effects were also not present. This was despite RT and variability in responding increasing with time-on-task—along with task-focus reports decreasing and mind-wandering reports increasing—indicating that people did become fatigued (Unsworth & Robison, 2016). The absence of any effect in the present study of background music on time-on-task effects—either on subjective report or RT data—suggests that background music does not make a difference to how fatigued people become. These findings are consistent with a study by Burkhard et al. (2018) that found that background music increased arousal but did not affect performance on a sustained-attention task.

Moreover, analysis of tempo, lyrics, and genre in the present study revealed no significant effects on RT, variability of RT, or indeed on thought-probe response proportions. These null results represent a failure to replicate past studies on tempo (e.g., Amezcua et al., 2005; Angel et al., 2010), lyrics (e.g., Darrow et al., 2006; Drai-Zerbib and Baccino, 2017; Shih et al., 2012) and on genre (e.g., Chew, 2010; Corhan and Gounard, 1976; Davies et al., 1973). The absence of any significant effect of musical parameters (whether tempo, lyrics, or genre) may be a by-product of the use of self-selected music in the current study, making it bound to represent a less satisfactory test of their role than studies directly manipulating these musical parameters within the individual. However, it is entirely possible that participants selected their background music to titrate their arousal to optimal levels, such that participants whose baseline arousal level was low selected more arousing music, while those whose baseline arousal level was high selected less arousing music; in this case, effects of musical parameters mediated by arousal would not be manifested. Because of the absence of an effect of tempo, lyrics, or genre in the current study, and the limited number of studies on these musicological parameters and sustained attention, future research could further explore whether these parameters exert significant effects when listening preferences are controlled for.

In summary, the current study demonstrated that preferred background music enhanced task-focus on a low-demanding sustained-attention task by decreasing mind-wandering; music did not affect external distraction at all. Furthermore, while on-task states were linked to shorter RT than off-task states, background music did not affect RT or variability of RT. Overall, these results provide evidence for a positive effect of background music on task-focused attention during an easy, low-demanding task. Future research should manipulate task-difficulty by including a difficult as well as an easy task. This should permit examination of the full range of the inverted-U curve linking arousal to performance and subjective attentional state, including both types of attentional lapses (mind-wandering and external distraction). The types of attentional lapses could also be broken down into more sub-types (e.g., to include task-relevant distractions, or more types of mind-wandering; see Unsworth & Robison, 2018) in future studies to advance our understanding of sub-optimal attentional states and the effect background music has on them. Moreover, to test the generalisability of our results, future experiments should include people who do not normally listen to background music. It would also be interesting to test whether our findings can be replicated when keeping the device for presentation of the sound stimuli (i.e., headphones) consistent across music-present and music-absent conditions.

## Conclusion

Listening to background music while performing tasks that require sustained attention is common, although previous research on the benefit of such music listening is inconsistent. The current study aimed to explore how the effects of preferred background music are mediated via arousal and how music affects attentional state. Using preferred or self-selected background music in the study increased ecological validity compared to the many studies investigating background music using researcher-selected music and also allowed us to take listening preferences and differences in baseline arousal levels into account. The present research is the first to investigate the effect of preferred background music on attentional states by collecting subjective reports as well as behavioural measures of performance (RT). The subjective reports employed here are founded on the inverted-U relationship between arousal and performance and can identify attentional lapses (i.e., mind-wandering, external distraction) as well as optimal states (i.e., task-focus), providing a richer and more informative index of attention than simple performance measures (RT). Importantly, listening to preferred background music was found to increase task-focus and decrease mind-wandering states on a low-demanding sustained-attention task although it did not affect overall RT. The increase in task-focus states provides evidence for music’s ability to improve focused attention and performance—by increasing arousal to an intermediate level optimal for performance—and suggests that people can derive benefit from music listening while performing low-demanding tasks. However, preferred background music did not affect time-on-task effects, which suggests that listening to music did not influence how fatigued people became over time.

## Data Availability

Datasets obtained during, and/or analysed during, the current study are available from the corresponding author on reasonable request.
